# Supplementary material for: Effective Approaches to Study the Genetic Variability of SARS-CoV-2
Source: Viruses. 2022 Aug 24;14(9):1855. doi: 10.3390/v14091855 (PMC9504788; doi:10.3390/v14091855)
Supplement: Supplementary file 1 [file viruses-14-01855-s001.zip › viruses-1876077-supplementary.pdf]

Effective approaches to study the genetic variability of SARS-CoV-2

Table S1

primer_name	primer_seq	pool
nCoV-2019_13_LEFT_Nextera	TCGTCGGCAGCGTCAGATGTGTATAAGAGACAGTCGCACAAATGTCTACTTAGCTGT	1
nCoV-2019_13_RIGHT_Nextera	GTCTCGTGGGCTCGGAGATGTGTATAAGAGACAGACCACAGCAGTTAAAACACCCT	1
nCoV-2019_98_LEFT_Nextera	TCGTCGGCAGCGTCAGATGTGTATAAGAGACAGAACAATTGCAACAATCCATGAGCA	1
nCoV-2019_98_RIGHT_Nextera	GTCTCGTGGGCTCGGAGATGTGTATAAGAGACAGTTCTCCTAAGAAGCTATTAAAATCACATGG	1
nCoV-2019_48_LEFT_Nextera	TCGTCGGCAGCGTCAGATGTGTATAAGAGACAGTGTTGACACTGACTTAACAAAGCCT	1
nCoV-2019_48_RIGHT_Nextera	GTCTCGTGGGCTCGGAGATGTGTATAAGAGACAGTAGATTACCAGAAGCAGCGTGC	1
nCoV-2019_36_LEFT_Nextera	TCGTCGGCAGCGTCAGATGTGTATAAGAGACAGTTAGCTTGGTTGTACGCTGCTG	1
nCoV-2019_36_RIGHT_Nextera	GTCTCGTGGGCTCGGAGATGTGTATAAGAGACAGGAACAAAGACCATTGAGTACTCTGGA	1
nCoV-2019_73_LEFT_Nextera	TCGTCGGCAGCGTCAGATGTGTATAAGAGACAGCAATTTTGTAATGATCCATTTTTGGGTGT	1
nCoV-2019_73_RIGHT_Nextera	GTCTCGTGGGCTCGGAGATGTGTATAAGAGACAGCACCAGCTGTCCAACCTGAAGA	1
nCoV-2019_93_LEFT_Nextera	TCGTCGGCAGCGTCAGATGTGTATAAGAGACAGTGAGGCTGGTTCTAAATCACCCA	1

nCoV-2019_93_RIGHT_Nextera	GTCTCGTGGGCTCGGAGATGTGTATAAGAGACAGAGGTCTTCCTTGCCATGTTGAG	1
nCoV-2019_90_LEFT_Nextera	TCGTCGGCAGCGTCAGATGTGTATAAGAGACAGACACAGACCATTCCAGTAGCAGT	1
nCoV-2019_90_RIGHT_Nextera	GTCTCGTGGGCTCGGAGATGTGTATAAGAGACAGTGAAATGGTGAATTGCCCTCGT	1
nCoV-2019_16_LEFT_Nextera	TCGTCGGCAGCGTCAGATGTGTATAAGAGACAGAATTTGGAAGAAGCTGCTCGGT	1
nCoV-2019_16_RIGHT_Nextera	GTCTCGTGGGCTCGGAGATGTGTATAAGAGACAGCACAACTTGCGTGTGGAGGTTA	1
nCoV-2019_86_LEFT_Nextera	TCGTCGGCAGCGTCAGATGTGTATAAGAGACAGTCAGGTGATGGCACAACAAGTC	1
nCoV-2019_86_RIGHT_Nextera	GTCTCGTGGGCTCGGAGATGTGTATAAGAGACAGACGAAAGCAAGAAAAAGAAGTACGC	1
nCoV-2019_39_LEFT_Nextera	TCGTCGGCAGCGTCAGATGTGTATAAGAGACAGAGTATTGCCCTATTTTCTTCATAACTGGT	1
nCoV-2019_39_RIGHT_Nextera	GTCTCGTGGGCTCGGAGATGTGTATAAGAGACAGTGTAACTGGACACATTGAGCCC	1
nCoV-2019_29_LEFT_Nextera	TCGTCGGCAGCGTCAGATGTGTATAAGAGACAGACTTGTGTTCCTTTTTGTTGCTGC	1
nCoV-2019_29_RIGHT_Nextera	GTCTCGTGGGCTCGGAGATGTGTATAAGAGACAGAGTGTACTCTATAAGTTTTGATGGTGTGT	1
nCoV-2019_79_LEFT_Nextera	TCGTCGGCAGCGTCAGATGTGTATAAGAGACAGGTGGTGATTCAACTGAATGCAGC	1
nCoV-2019_79_RIGHT_Nextera	GTCTCGTGGGCTCGGAGATGTGTATAAGAGACAGCATTTCATCTGTGAGCAAAGGTGG	1

SARS-CoV-2_1_LEFTv4	TCGTCGGCAGCGTCAGATGTGTATAAGAGACAGAACAAACCAACCAACTTTCGATCTC	1
SARS-CoV-2_1_RIGHTv4	GTCTCGTGGGCTCGGAGATGTGTATAAGAGACAGCTTCTACTAAGCCACAAGTGCCA	1
nCoV-2019_27_LEFT_Nextera	TCGTCGGCAGCGTCAGATGTGTATAAGAGACAGACTACAGTCAGCTTATGTGTCAACC	1
nCoV-2019_27_RIGHT_Nextera	GTCTCGTGGGCTCGGAGATGTGTATAAGAGACAGAATACAAGCACCAAGGTCACGG	1
nCoV-2019_54_LEFT_Nextera	TCGTCGGCAGCGTCAGATGTGTATAAGAGACAGTGAGTTAACAGGACACATGTTAGACA	1
nCoV-2019_54_RIGHT_Nextera	GTCTCGTGGGCTCGGAGATGTGTATAAGAGACAGAACCAAAAACTTGTCCATTAGCACA	1
nCoV-2019_33_LEFT_Nextera	TCGTCGGCAGCGTCAGATGTGTATAAGAGACAGACTTTTGAAGAAGCTGCGCTGT	1
nCoV-2019_33_RIGHT_Nextera	GTCTCGTGGGCTCGGAGATGTGTATAAGAGACAGTGGACAGTAAACTACGTCATCAAGC	1
nCoV-2019_3_LEFT_Nextera	TCGTCGGCAGCGTCAGATGTGTATAAGAGACAGCGGTAATAAAGGAGCTGGTGGC	1
nCoV-2019_3_RIGHT_Nextera	GTCTCGTGGGCTCGGAGATGTGTATAAGAGACAGAAGGTGTCTGCAATTCATAGCTCT	1
nCoV-2019_68_LEFT_Nextera	TCGTCGGCAGCGTCAGATGTGTATAAGAGACAGACAGGTTCATCTAAGTGTGTGTGT	2
nCoV-2019_68_RIGHT_Nextera	GTCTCGTGGGCTCGGAGATGTGTATAAGAGACAGCTCCTTTATCAGAACCAGCACCA	2
nCoV-2019_56_LEFT_Nextera	TCGTCGGCAGCGTCAGATGTGTATAAGAGACAGACCTAGACCACCACTTAACCGA	2
nCoV-2019_56_RIGHT_Nextera	GTCTCGTGGGCTCGGAGATGTGTATAAGAGACAGACACTATGCGAGCAGAAGGGTA	2

nCoV-2019_74_LEFT_Nextera	TCGTCGGCAGCGTCAGATGTGTATAAGAGACAGACATCACTAGGTTTCAAACTTTACTTGC	2
nCoV-2019_74_RIGHT_Nextera	GTCTCGTGGGCTCGGAGATGTGTATAAGAGACAGGCAACACAGTTGCTGATTCTCTTC	2
nCoV-2019_78_LEFT_Nextera	TCGTCGGCAGCGTCAGATGTGTATAAGAGACAGCAACTTACTCCTACTTGGCGTGT	2
nCoV-2019_78_RIGHT_Nextera	GTCTCGTGGGCTCGGAGATGTGTATAAGAGACAGTGTGTACAAAAACTGCCATATTGCA	2
nCoV-2019_43_LEFT_Nextera_v2	TCGTCGGCAGCGTCAGATGTGTATAAGAGACAGATTGTTCAACTTAGTGAAATTAGTATGGAC	2
nCoV-2019_43_RIGHT_Nextera	GTCTCGTGGGCTCGGAGATGTGTATAAGAGACAGAGCAGCATCTACAGCAAAAGCA	2
nCoV-2019_63_LEFT_Nextera	TCGTCGGCAGCGTCAGATGTGTATAAGAGACAGTGTTAAGCGTGTTGACTGGACT	2
nCoV-2019_63_RIGHT_Nextera	GTCTCGTGGGCTCGGAGATGTGTATAAGAGACAGACAAACTGCCACCATCACAACC	2
nCoV-2019_66_LEFT_Nextera	TCGTCGGCAGCGTCAGATGTGTATAAGAGACAGGGGTGTGGACATTGCTGCTAAT	2
nCoV-2019_66_RIGHT_Nextera	GTCTCGTGGGCTCGGAGATGTGTATAAGAGACAGTCAATTTCCATTTGACTCCTGGGT	2
nCoV-2019_5_LEFT_Nextera	TCGTCGGCAGCGTCAGATGTGTATAAGAGACAGTGGTGAAACTTCATGGCAGACG	2
nCoV-2019_5_RIGHT_Nextera	GTCTCGTGGGCTCGGAGATGTGTATAAGAGACAGATTGATGTTGACTTTCTCTTTTTGGAGT	2
nCoV-2019_49_LEFT_Nextera_v2	TCGTCGGCAGCGTCAGATGTGTATAAGAGACAGCATTGTGCAAACTTTAATGTTTTATTC	2

nCoV-2019_49_RIGHT_Nextera	GTCTCGTGGGCTCGGAGATGTGTATAAGAGACAGTGACGATGACTTGGTTAGCATTAATACA	2
nCoV-2019_12_LEFT_Nextera	TCGTCGGCAGCGTCAGATGTGTATAAGAGACAGAAACATGGAGGAGGTGTTGCAG	2
nCoV-2019_12_RIGHT_Nextera	GTCTCGTGGGCTCGGAGATGTGTATAAGAGACAGTTCACTCTTCATTTCCAAAAAGCTTGA	2
nCoV-2019_22_LEFT_Nextera	TCGTCGGCAGCGTCAGATGTGTATAAGAGACAGACTACCGAAGTTGTAGGAGACATTATACT	2
nCoV-2019_22_RIGHT_Nextera	GTCTCGTGGGCTCGGAGATGTGTATAAGAGACAGACAGTATTCTTTGCTATAGTAGTCGGC	2
nCoV-2019_53_LEFT_Nextera	TCGTCGGCAGCGTCAGATGTGTATAAGAGACAGAGCAAAATGTTGGACTGAGACTGA	2
nCoV-2019_53_RIGHT_Nextera	GTCTCGTGGGCTCGGAGATGTGTATAAGAGACAGAGCCTCATAAAACTCAGGTTCCC	2
nCoV-2019_58_LEFT_Nextera	TCGTCGGCAGCGTCAGATGTGTATAAGAGACAGTGATTTGAGTGTTGTCAATGCCAGA	2
nCoV-2019_58_RIGHT_Nextera	GTCTCGTGGGCTCGGAGATGTGTATAAGAGACAGCTTTTCTCCAAGCAGGGTTACGT	2
nCoV-2019_84_LEFT_Nextera	TCGTCGGCAGCGTCAGATGTGTATAAGAGACAGTGCTGTAGTTGTCTCAAGGGCT	2
nCoV-2019_84_RIGHT_Nextera	GTCTCGTGGGCTCGGAGATGTGTATAAGAGACAGAGGTGTGAGTAAACTGTTACAAACAAC	2
nCoV-2019_81_LEFT_Nextera	TCGTCGGCAGCGTCAGATGTGTATAAGAGACAGGCACTTGGAAAACTTCAAGATGTGG	2
nCoV-2019_81_RIGHT_Nextera	GTCTCGTGGGCTCGGAGATGTGTATAAGAGACAGGTGAAGTTCTTTTCTTGTGCAGGG	2

nCoV-2019_41_LEFT_Nextera	TCGTCGGCAGCGTCAGATGTGTATAAGAGACAGGTTCCCTTCCATCATATGCAGCT	2
nCoV-2019_41_RIGHT_Nextera	GTCTCGTGGGCTCGGAGATGTGTATAAGAGACAGTGGTATGACAACCATTAGTTTGGCT	2
nCoV-2019_45_LEFT_Nextera	TCGTCGGCAGCGTCAGATGTGTATAAGAGACAGTACCTACAACTTGTGCTAATGACCC	2
nCoV-2019_45_RIGHT_Nextera	GTCTCGTGGGCTCGGAGATGTGTATAAGAGACAGAAATTGTTTCTTCATGTTGGTAGTTAGAGA	2
nCoV-2019_17_LEFT_Nextera	TCGTCGGCAGCGTCAGATGTGTATAAGAGACAGCTTCTTTCTTTGAGAGAAGTGAGGACT	2
nCoV-2019_17_RIGHT_Nextera	GTCTCGTGGGCTCGGAGATGTGTATAAGAGACAGTTTGTTGGAGTGTTAACAATGCAGT	2
nCoV-2019_96_LEFT_Nextera_v2	TCGTCGGCAGCGTCAGATGTGTATAAGAGACAGTTCTCGTTCCTCATCACGTAG	2
nCoV-2019_96_RIGHT_Nextera	GTCTCGTGGGCTCGGAGATGTGTATAAGAGACAGTAGGCTCTGTTGGTGGGAATGT	2
nCoV-2019_70_LEFT_Nextera	TCGTCGGCAGCGTCAGATGTGTATAAGAGACAGACAAAAGAAAATGACTCTAAAGAGGGTTT	2
nCoV-2019_70_RIGHT_Nextera	GTCTCGTGGGCTCGGAGATGTGTATAAGAGACAGTGACCTTCTTTTAAAGACATAACAGCAG	2
nCoV-2019_9_LEFT_Nextera	TCGTCGGCAGCGTCAGATGTGTATAAGAGACAGTCCCACAGAAGTGTTAACAGAGGA	3
nCoV-2019_9_RIGHT_Nextera	GTCTCGTGGGCTCGGAGATGTGTATAAGAGACAGATGACAGCATCTGCCACAACAC	3
nCoV-2019_38_LEFT_Nextera	TCGTCGGCAGCGTCAGATGTGTATAAGAGACAGACTGTGTTATGTATGCATCAGCTGT	3

NcoV-2019_38_RIGHT_Nextera_v2	GTCTCGTGGGCTCGGAGATGTGTATAAGAGACAGCCAACAATTTAATGTTGAGTTTGAAG	3
nCoV-2019_23_LEFT_Nextera_v2	TCGTCGGCAGCGTCAGATGTGTATAAGAGACAGAACCCTTGCTACTCATGGTTTAG	3
nCoV-2019_23_RIGHT_Nextera	GTCTCGTGGGCTCGGAGATGTGTATAAGAGACAGACCAGTACAGTAGGTTGCAATAGTG	3
nCoV-2019_30_LEFT_Nextera	TCGTCGGCAGCGTCAGATGTGTATAAGAGACAGGCACAACTAATGGTGACTTTTTGCA	3
nCoV-2019_30_RIGHT_Nextera	GTCTCGTGGGCTCGGAGATGTGTATAAGAGACAGACCACTAGTAGATACACAAACACCAG	3
nCoV-2019_94_LEFT_Nextera	TCGTCGGCAGCGTCAGATGTGTATAAGAGACAGGGCCCCAAGGTTTACCCAATAA	3
nCoV-2019_94_RIGHT_Nextera	GTCTCGTGGGCTCGGAGATGTGTATAAGAGACAGTTTGGCAATGTTGTTCCTTGAGG	3
nCoV-2019_75_LEFT_Nextera	TCGTCGGCAGCGTCAGATGTGTATAAGAGACAGAGAGTCCAACCAACAGAATCTATTGT	3
nCoV-2019_75_RIGHT_Nextera	GTCTCGTGGGCTCGGAGATGTGTATAAGAGACAGACCACCAACCTTAGAATCAAGATTGT	3
nCoV-2019_59_LEFT_Nextera	TCGTCGGCAGCGTCAGATGTGTATAAGAGACAGTCACGCATGATGTTTCATCTGCA	3
nCoV-2019_59_RIGHT_Nextera	GTCTCGTGGGCTCGGAGATGTGTATAAGAGACAGAAGAGTCCTGTTACATTTTCAGCTTG	3
nCoV-2019_25_LEFT_Nextera	TCGTCGGCAGCGTCAGATGTGTATAAGAGACAGGCAATTGTTTTTCAGCTATTTTGCAGT	3
nCoV-2019_25_RIGHT_Nextera	GTCTCGTGGGCTCGGAGATGTGTATAAGAGACAGACTGTAGTGACAAGTCTCTCGCA	3

nCoV-2019_57_LEFT_Nextera	TCGTCGGCAGCGTCAGATGTGTATAAGAGACAGATTCTACACTCCAGGGACCACC	3
nCoV-2019_57_RIGHT_Nextera	GTCTCGTGGGCTCGGAGATGTGTATAAGAGACAGGTAATTGAGCAGGGTCGCCAAT	3
nCoV-2019_85_LEFT_Nextera	TCGTCGGCAGCGTCAGATGTGTATAAGAGACAGACTAGCACTCTCCAAGGGTGTT	3
nCoV-2019_85_RIGHT_Nextera	GTCTCGTGGGCTCGGAGATGTGTATAAGAGACAGACACAGTCTTTTACTCCAGATTCCC	3
nCoV-2019_52_LEFT_Nextera	TCGTCGGCAGCGTCAGATGTGTATAAGAGACAGCATCAGGAGATGCCACAACTGC	3
nCoV-2019_52_RIGHT_Nextera	GTCTCGTGGGCTCGGAGATGTGTATAAGAGACAGGTTGAGAGCAAAATTCATGAGGTCC	3
nCoV-2019_55_LEFT_Nextera	TCGTCGGCAGCGTCAGATGTGTATAAGAGACAGACTCAACTTTACTTAGGAGGTATGAGCT	3
nCoV-2019_55_RIGHT_Nextera	GTCTCGTGGGCTCGGAGATGTGTATAAGAGACAGGGTGTACTCTCCTATTTGTACTTTACTGT	3
nCoV-2019_40_LEFT_Nextera	TCGTCGGCAGCGTCAGATGTGTATAAGAGACAGTGCACATCAGTAGTCTTACTCTCAGT	3
nCoV-2019_40_RIGHT_Nextera	GTCTCGTGGGCTCGGAGATGTGTATAAGAGACAGCATGGCTGCATCACGGTCAAAT	3
nCoV-2019_83_LEFT_Nextera	TCGTCGGCAGCGTCAGATGTGTATAAGAGACAGTCCTTTGCAACCTGAATTAGACTCA	3
nCoV-2019_83_RIGHT_Nextera	GTCTCGTGGGCTCGGAGATGTGTATAAGAGACAGTTTGACTCCTTTGAGCACTGGC	3
nCoV-2019_50_LEFT_alt_Nextera_v3	TCGTCGGCAGCGTCAGATGTGTATAAGAGACAGAATTAAAACACTTCTTCTTTGCTCAG	3

nCoV-2019_50_RIGHT_v2_Nextera	GTCTCGTGGGCTCGGAGATGTGTATAAGAGACAGAGGCATGGCTCTATCACATTTAG	3
nCoV-2019_20_LEFT_Nextera	TCGTCGGCAGCGTCAGATGTGTATAAGAGACAGACAAAGAAAACAGTTACACAACAACCA	3
nCoV-2019_20_RIGHT_Nextera	GTCTCGTGGGCTCGGAGATGTGTATAAGAGACAGACGTGGCTTTATTAGTTGCATTGTT	3
nCoV-2019_47_LEFT_Nextera	TCGTCGGCAGCGTCAGATGTGTATAAGAGACAGAGGACTGGTATGATTTTGTAGAAAACCC	3
nCoV-2019_47_RIGHT_Nextera	GTCTCGTGGGCTCGGAGATGTGTATAAGAGACAGAATAACGGTCAAAGAGTTTTAACCTCTC	3
nCoV-2019_32_LEFT_Nextera	TCGTCGGCAGCGTCAGATGTGTATAAGAGACAGTGGTGAATACAGTCATGTAGTTGCC	3
nCoV-2019_32_RIGHT_Nextera	GTCTCGTGGGCTCGGAGATGTGTATAAGAGACAGAGCACATCACTACGCAACTTTAGA	3
nCoV-2019_14_LEFT_Nextera	TCGTCGGCAGCGTCAGATGTGTATAAGAGACAGCATCCAGATTCTGCCACTCTTGT	3
nCoV-2019_14_RIGHT_Nextera	GTCTCGTGGGCTCGGAGATGTGTATAAGAGACAGAGTTTCCACACAGACAGGCATT	3
NcoV-2019_72_LEFT_Nextera_v2	TCGTCGGCAGCGTCAGATGTGTATAAGAGACAGACCAGAACTCAATTACCCCC	3
nCoV-2019_72_RIGHT_Nextera_v2	GTCTCGTGGGCTCGGAGATGTGTATAAGAGACAGCAATATTCTTAAACACAAATTCCCTAAG	3
nCoV-2019_18_LEFT_alt2_Nextera	TCGTCGGCAGCGTCAGATGTGTATAAGAGACAGACTTCTATTAAATGGGCAGATAACAACTGT	3
nCoV-2019_18_RIGHT_alt1_Nextera	GTCTCGTGGGCTCGGAGATGTGTATAAGAGACAGGCTTGTTTACCACACGTACAAGG	3

nCoV-2019_46_LEFT_alt1_Nextera	TCGTCGGCAGCGTCAGATGTGTATAAGAGACAGCGCTTCCAAGAAAAGGACGAAGA	4
nCoV-2019_46_RIGHT_alt2_Nextera	GTCTCGTGGGCTCGGAGATGTGTATAAGAGACAGCACGTTCACCTAAGTTGGCGTAT	4
nCoV-2019_92_LEFT_Nextera	TCGTCGGCAGCGTCAGATGTGTATAAGAGACAGTTTGTGCTTTTTAGCCTTTCTGCT	4
nCoV-2019_92_RIGHT_Nextera	GTCTCGTGGGCTCGGAGATGTGTATAAGAGACAGAGGTTCCTGGCAATTAATTGTAAAAGG	4
nCoV-2019_28_LEFT_Nextera	TCGTCGGCAGCGTCAGATGTGTATAAGAGACAGACATAGAAGTTACTGGCGATAGTTGT	4
nCoV-2019_28_RIGHT_Nextera	GTCTCGTGGGCTCGGAGATGTGTATAAGAGACAGTGTTTAGACATGACATGAACAGGTGT	4
nCoV-2019_35_LEFT_Nextera	TCGTCGGCAGCGTCAGATGTGTATAAGAGACAGTGTTCGCATTCAACCAGGACAG	4
nCoV-2019_35_RIGHT_Nextera	GTCTCGTGGGCTCGGAGATGTGTATAAGAGACAGACTTCATAGCCACAAGGTTAAAGTCA	4
nCoV-2019_44_LEFT_alt3_Nextera	TCGTCGGCAGCGTCAGATGTGTATAAGAGACAGCCACAGTACGTCTACAAGCTGG	4
nCoV-2019_44_RIGHT_alt0_Nextera	GTCTCGTGGGCTCGGAGATGTGTATAAGAGACAGCGCAGACGGTACAGACTGTGTT	4
nCoV-2019_60_LEFT_Nextera	TCGTCGGCAGCGTCAGATGTGTATAAGAGACAGTGATAGAGACCTTTATGACAAGTTGCA	4
nCoV-2019_60_RIGHT_Nextera	GTCTCGTGGGCTCGGAGATGTGTATAAGAGACAGGGTACCAACAGCTTCTCTAGTAGC	4
nCoV-2019_26_LEFT_Nextera	TCGTCGGCAGCGTCAGATGTGTATAAGAGACAGTTGTGATACATTCTGTGCTGGTAGT	4

nCoV-2019_26_RIGHT_Nextera	GTCTCGTGGGCTCGGAGATGTGTATAAGAGACAGTCCGCACTATCACCAACATCAG	4
nCoV-2019_31_LEFT_Nextera	TCGTCGGCAGCGTCAGATGTGTATAAGAGACAGTTCTGAGTACTGTAGGCACGGC	4
nCoV-2019_31_RIGHT_Nextera	GTCTCGTGGGCTCGGAGATGTGTATAAGAGACAGACAGAATAAACACCAGGTAAGAATGAGT	4
nCoV-2019_76_LEFT_Nextera	TCGTCGGCAGCGTCAGATGTGTATAAGAGACAGAGGGCAAACTGGAAAGATTGCT	4
nCoV-2019_76_RIGHT_alt0_Nextera	GTCTCGTGGGCTCGGAGATGTGTATAAGAGACAGACCTGTGCCTGTTAAACCATTGA	4
nCoV-2019_37_LEFT_Nextera	TCGTCGGCAGCGTCAGATGTGTATAAGAGACAGACACACCACTGGTTGTTACTCAC	4
nCoV-2019_37_RIGHT_Nextera	GTCTCGTGGGCTCGGAGATGTGTATAAGAGACAGGTCCACACTCTCCTAGCACCAT	4
nCoV-2019_15_LEFT_alt1_Nextera	TCGTCGGCAGCGTCAGATGTGTATAAGAGACAGAGTGCTTAAAAAGTGTAAAAGTGCCT	4
nCoV-2019_15_RIGHT_alt3_Nextera	GTCTCGTGGGCTCGGAGATGTGTATAAGAGACAGACTGTAGCTGGCACTTTGAGAGA	4
nCoV-2019_62_LEFT_Nextera	TCGTCGGCAGCGTCAGATGTGTATAAGAGACAGGGCACATGGCTTTGAGTTGACA	4
nCoV-2019_62_RIGHT_Nextera	GTCTCGTGGGCTCGGAGATGTGTATAAGAGACAGGTTGAACCTTTCTACAAGCCGC	4
nCoV-2019_21_LEFT_alt2_Nextera	TCGTCGGCAGCGTCAGATGTGTATAAGAGACAGGGCTATTGATTATAAACACTACACACCCT	4
nCoV-2019_21_RIGHT_alt0_Nextera	GTCTCGTGGGCTCGGAGATGTGTATAAGAGACAGGATCTGTGTGGCCAACCTCTTC	4

nCoV-2019_19_LEFT_Nextera	TCGTCGGCAGCGTCAGATGTGTATAAGAGACAGGCTGTTATGTACATGGGCACACT	4
nCoV-2019_19_RIGHT_Nextera	GTCTCGTGGGCTCGGAGATGTGTATAAGAGACAGTGTCCAACTTAGGGTCAATTTCTGT	4
nCoV-2019_97_LEFT_Nextera	TCGTCGGCAGCGTCAGATGTGTATAAGAGACAGTGGATGACAAAGATCCAAATTTCAAAGA	4
nCoV-2019_97_RIGHT_Nextera_v2	GTCTCGTGGGCTCGGAGATGTGTATAAGAGACAGTTTACACATTAGGGCTCTTCCATATAG	4
nCoV-2019_8_LEFT_Nextera	TCGTCGGCAGCGTCAGATGTGTATAAGAGACAGAGAGTTTCTTAGAGACGGTTGGGA	4
nCoV-2019_8_RIGHT_Nextera	GTCTCGTGGGCTCGGAGATGTGTATAAGAGACAGGCTTCAACAGCTTCACTAGTAGGT	4
nCoV-2019_6_LEFT_Nextera	TCGTCGGCAGCGTCAGATGTGTATAAGAGACAGGGTGTTGTTGGAGAAGGTTCCG	4
nCoV-2019_6_RIGHT_Nextera	GTCTCGTGGGCTCGGAGATGTGTATAAGAGACAGTAGCGGCCTTCTGTAAAACACG	4
nCoV-2019_42_LEFT_Nextera	TCGTCGGCAGCGTCAGATGTGTATAAGAGACAGTGCAAGAGATGGTTGTGTTCCC	4
nCoV-2019_42_RIGHT_Nextera	GTCTCGTGGGCTCGGAGATGTGTATAAGAGACAGCCTACCTCCCTTTGTTGTGTTGT	4
nCoV-2019_88_LEFT_Nextera	TCGTCGGCAGCGTCAGATGTGTATAAGAGACAGCCATGGCAGATTCCAACGGTAC	4
nCoV-2019_88_RIGHT_Nextera	GTCTCGTGGGCTCGGAGATGTGTATAAGAGACAGTGGTCAGAATAGTGCCATGGAGT	4
nCoV-2019_11_LEFT_Nextera	TCGTCGGCAGCGTCAGATGTGTATAAGAGACAGGGAATTTGGTGCCACTTCTGCT	4

nCoV-2019_11_RIGHT_Nextera	GTCTCGTGGGCTCGGAGATGTGTATAAGAGACAGTCATCAGATTCAACTTGCATGGCA	4
nCoV-2019_65_LEFT_Nextera	TCGTCGGCAGCGTCAGATGTGTATAAGAGACAGGCTGGCTTTAGCTTGTGGGTTT	4
nCoV-2019_65_RIGHT_Nextera	GTCTCGTGGGCTCGGAGATGTGTATAAGAGACAGTGTCAGTCATAGAACAAACACCAATAGT	4
nCoV-2019_24_LEFT_Nextera	TCGTCGGCAGCGTCAGATGTGTATAAGAGACAGAGGCATGCCTTCTTACTGTACTG	5
nCoV-2019_24_RIGHT_Nextera	GTCTCGTGGGCTCGGAGATGTGTATAAGAGACAGACATTCTAACCATAGCTGAAATCGGG	5
nCoV-2019_80_LEFT_Nextera	TCGTCGGCAGCGTCAGATGTGTATAAGAGACAGTTGCCTTGGTGATATTGCTGCT	5
nCoV-2019_80_RIGHT_Nextera	GTCTCGTGGGCTCGGAGATGTGTATAAGAGACAGTGGAGCTAAGTTGTTTAACAAGCG	5
nCoV-2019_71_LEFT_Nextera	TCGTCGGCAGCGTCAGATGTGTATAAGAGACAGACAAATCCAATTCAGTTGTCTTCCTATTC	5
nCoV-2019_71_RIGHT_Nextera	GTCTCGTGGGCTCGGAGATGTGTATAAGAGACAGTGGAAAAGAAAGGTAAGAACAAGTCCT	5
nCoV-2019_4_LEFT_Nextera	TCGTCGGCAGCGTCAGATGTGTATAAGAGACAGGGTGTATACTGCTGCCGTGAAC	5
nCoV-2019_4_RIGHT_Nextera	GTCTCGTGGGCTCGGAGATGTGTATAAGAGACAGCACAAGTAGTGGCACCTTCTTTAGT	5
nCoV-2019_91_LEFT_Nextera	TCGTCGGCAGCGTCAGATGTGTATAAGAGACAGTCACTACCAAGAGTGTGTTAGAGGT	5
nCoV-2019_91_RIGHT_Nextera	GTCTCGTGGGCTCGGAGATGTGTATAAGAGACAGTTCAAGTGAGAACCAAAAGATAATAAGCA	5

nCoV-2019_51_LEFT_Nextera	TCGTCGGCAGCGTCAGATGTGTATAAGAGACAGTCAATAGCCGCCACTAGAGGAG	5
nCoV-2019_51_RIGHT_Nextera	GTCTCGTGGGCTCGGAGATGTGTATAAGAGACAGAGTGCATTAACATTGGCCGTGA	5
nCoV-2019_87_LEFT_Nextera	TCGTCGGCAGCGTCAGATGTGTATAAGAGACAGCGACTACTAGCGTGCCTTTGTA	5
nCoV-2019_87_RIGHT_Nextera	GTCTCGTGGGCTCGGAGATGTGTATAAGAGACAGACTAGGTTCCATTGTTCAAGGAGC	5
nCoV-2019_64_LEFT_Nextera_v2	TCGTCGGCAGCGTCAGATGTGTATAAGAGACAGGCCACACATTCTGACAAATTC	5
nCoV-2019_64_RIGHT_Nextera	GTCTCGTGGGCTCGGAGATGTGTATAAGAGACAGAGTCTTGTAAAAGTGTTCCAGAGGT	5
nCoV-2019_95_LEFT_Nextera	TCGTCGGCAGCGTCAGATGTGTATAAGAGACAGTGAGGGAGCCTTGAATACACCA	5
nCoV-2019_95_RIGHT_Nextera	GTCTCGTGGGCTCGGAGATGTGTATAAGAGACAGCAGTACGTTTTTGCCGAGGCTT	5
nCoV-2019_2_LEFT_Nextera	TCGTCGGCAGCGTCAGATGTGTATAAGAGACAGCTGTTTTACAGGTTCGCGACGT	5
nCoV-2019_2_RIGHT_Nextera	GTCTCGTGGGCTCGGAGATGTGTATAAGAGACAGTAAGGATCAGTGCCAAGCTCGT	5
nCoV-2019_10_LEFT_Nextera	TCGTCGGCAGCGTCAGATGTGTATAAGAGACAGTGAGAAGTGCTCTGCCTATACAGT	5
nCoV-2019_10_RIGHT_Nextera	GTCTCGTGGGCTCGGAGATGTGTATAAGAGACAGTCATCTAACCAATCTTCTTCTTGCTCT	5
nCoV-2019_77_LEFT_Nextera	TCGTCGGCAGCGTCAGATGTGTATAAGAGACAGCCAGCAACTGTTTGTGGACCTA	5

nCoV-2019_77_RIGHT_Nextera	GTCTCGTGGGCTCGGAGATGTGTATAAGAGACAGCAGCCCCTATTAAACAGCCTGC	5
nCoV-2019_67_LEFT_Nextera	TCGTCGGCAGCGTCAGATGTGTATAAGAGACAGGTTGTCCAACAATTACCTGAAACTTACT	5
nCoV-2019_67_RIGHT_Nextera	GTCTCGTGGGCTCGGAGATGTGTATAAGAGACAGCAACCTTAGAAACTACAGATAAATCTTGGG	5
nCoV-2019_82_LEFT_Nextera	TCGTCGGCAGCGTCAGATGTGTATAAGAGACAGGGGCTATCATCTTATGTCCTTCCCT	5
nCoV-2019_82_RIGHT_Nextera	GTCTCGTGGGCTCGGAGATGTGTATAAGAGACAGTGCCAGAGATGTCACCTAAATCAA	5
nCoV-2019_89_LEFT_Nextera	TCGTCGGCAGCGTCAGATGTGTATAAGAGACAGGTACGCGTTCCATGTGGTCATT	5
nCoV-2019_89_RIGHT_Nextera	GTCTCGTGGGCTCGGAGATGTGTATAAGAGACAGACCTGAAAGTCAACGAGATGAAACA	5
nCoV-2019_69_LEFT_Nextera	TCGTCGGCAGCGTCAGATGTGTATAAGAGACAGTGTCGCAAAATATACTCAACTGTGTCA	5
nCoV-2019_69_RIGHT_Nextera	GTCTCGTGGGCTCGGAGATGTGTATAAGAGACAGTCTTTATAGCCACGGAACCTCCA	5
nCoV-2019_61_LEFT_Nextera	TCGTCGGCAGCGTCAGATGTGTATAAGAGACAGTGTTTATCACCCGCGAAGAAGC	5
nCoV-2019_61_RIGHT_Nextera	GTCTCGTGGGCTCGGAGATGTGTATAAGAGACAGATCACATAGACAACAGGTGCGC	5
nCoV-2019_7_LEFT_Nextera	TCGTCGGCAGCGTCAGATGTGTATAAGAGACAGATCAGAGGCTGCTCGTGTTGTA	5
nCoV-2019_7_RIGHT_Nextera	GTCTCGTGGGCTCGGAGATGTGTATAAGAGACAGTGCACAGGTGACAATTTGTCCA	5

nCoV-2019_34_LEFT_Nextera	TCGTCGGCAGCGTCAGATGTGTATAAGAGACAGTCCCATCTGGTAAAGTTGAGGGT	5
nCoV-2019_34_RIGHT_Nextera	GTCTCGTGGGCTCGGAGATGTGTATAAGAGACAGAGTGAAATTGGGCCTCATAGCA	5

Table S2

**primer_name**	**primer_seq**	**pool**
S-L1	TCGTCGGCAGCGTCAGATGTGTATAAGAGACAGTTATTGCCACTAGTCTCTAGTCAGTGTG	1
S-R1	GTCTCGTGGGCTCGGAGATGTGTATAAGAGACAGCATTGGAAAAGAAAGGTAAGAACAAG	1
S-L4	TCGTCGGCAGCGTCAGATGTGTATAAGAGACAGCTCTCAGCCTTTTCTTATGGACC	1
S-R4	GTCTCGTGGGCTCGGAGATGTGTATAAGAGACAGCAGGAGTCAAATAACTTCTATGTAAAGCAAG	1
S-L10	TCGTCGGCAGCGTCAGATGTGTATAAGAGACAGGGTTTAACAGGCACAGGTGTTC	1
S-R10	GTCTCGTGGGCTCGGAGATGTGTATAAGAGACAGAACACGCCAAGTAGGAGTAAGTTG	1
S-L13	TCGTCGGCAGCGTCAGATGTGTATAAGAGACAGCAGTGTCTATGACCAAGACATCAGTAG	1
S-R13	GTCTCGTGGGCTCGGAGATGTGTATAAGAGACAGGTTTTGATGGATCTGGTAATATTTGTG	1
S-L16	TCGTCGGCAGCGTCAGATGTGTATAAGAGACAGAAACTTCAAGATGTGGTCAACCA	1
S-R16	GTCTCGTGGGCTCGGAGATGTGTATAAGAGACAGGAAGCTCTGATTTCTGCAGCTC	1
S-L2	TCGTCGGCAGCGTCAGATGTGTATAAGAGACAGTCAGATCCTCAGTTTTACATTCAACTC	2
S-R2	GTCTCGTGGGCTCGGAGATGTGTATAAGAGACAGAGGGACTGGGTCTTCGAATC	2
S-L5	TCGTCGGCAGCGTCAGATGTGTATAAGAGACAGGCCAATAGGTATTAACATCACTAGGTTTC	2
S-R5	GTCTCGTGGGCTCGGAGATGTGTATAAGAGACAGTGGTTGGACTCTAAAGTTAGAAGTTTGATAG	2

S-L8	TCGTCGGCAGCGTCAGATGTGTATAAGAGACAGCAGGCTGCGTTATAGCTTGG	2
S-R8	GTCTCGTGGGCTCGGAGATGTGTATAAGAGACAGCAAACAGTTGCTGGTGCATG	2
S-L11	TCGTCGGCAGCGTCAGATGTGTATAAGAGACAGCTTCTAACCAGGTTGCTGTTCTTTATC	2
S-R11	GTCTCGTGGGCTCGGAGATGTGTATAAGAGACAGACCAAGTGACATAGTGTAGGCAATG	2
S-L17	TCGTCGGCAGCGTCAGATGTGTATAAGAGACAGAATTGATAGGTTGATCACAGGCAG	2
S-R17	GTCTCGTGGGCTCGGAGATGTGTATAAGAGACAGTTTTCCATCATGACAAATGGC	2
S-L19	TCGTCGGCAGCGTCAGATGTGTATAAGAGACAGTGTCTGGTAACTGTGATGTTGTAATAGG	2
S-R19	GTCTCGTGGGCTCGGAGATGTGTATAAGAGACAGCAAGTTCTTGGAGATCGATGAGAG	2
S-L3	TCGTCGGCAGCGTCAGATGTGTATAAGAGACAGACATAATAAGAGGCTGGATTTTTGG	3
S-R3	GTCTCGTGGGCTCGGAGATGTGTATAAGAGACAGAGATTTTTGAAATTACCCTGTTTTCC	3
S-L6	TCGTCGGCAGCGTCAGATGTGTATAAGAGACAGTTGAAATCCTTCACTGTAGAAAAAGG	3
S-R6	GTCTCGTGGGCTCGGAGATGTGTATAAGAGACAGTCTGCATAGACATTAGTAAAGCAGAGATC	3
S-L9_alt1	TCGTCGGCAGCGTCAGATGTGTATAAGAGACAGTAATGGTGTTGCAGGTTTTAATTG	3
S-R9_alt1	GTCTCGTGGGCTCGGAGATGTGTATAAGAGACAGAAATTGTTGGAAAGGCAGAAAC	3
S-L12	TCGTCGGCAGCGTCAGATGTGTATAAGAGACAGCGGGCACGTAGTGTAGCTAGTC	3
S-R12	GTCTCGTGGGCTCGGAGATGTGTATAAGAGACAGTCAACAGCTATTCCAGTTAAAGCAC	3
S-L15	TCGTCGGCAGCGTCAGATGTGTATAAGAGACAGTCACAGATGAAATGATTGCTCAATAC	3
S-R15	GTCTCGTGGGCTCGGAGATGTGTATAAGAGACAGCAAGCGTGTTTAAAGCTTGTGC	3

S-L18	TCGTCGGCAGCGTCAGATGTGTATAAGAGACAGCATGGTGTAGTCTTCTTGCATGTG	3
S-R18	GTCTCGTGGGCTCGGAGATGTGTATAAGAGACAGTGCAAAGGATCATAAACTGTGTTG	3
S-L20	TCGTCGGCAGCGTCAGATGTGTATAAGAGACAGTTCAAAAAGAAATTGACCGCC	3
S-R20	GTCTCGTGGGCTCGGAGATGTGTATAAGAGACAGTTGAGACAACTACAGCAACTGGTC	3
S-L7_alt1	TCGTCGGCAGCGTCAGATGTGTATAAGAGACAGTTTTCACTTTTAAGTGTTATGGAGTGTCT	4
S-R7_alt1	GTCTCGTGGGCTCGGAGATGTGTATAAGAGACAGCCACTAACCTTAGAATCAAGCTTG	4
S-L14	TCGTCGGCAGCGTCAGATGTGTATAAGAGACAGCCACCAATTAAAGATTTTGGTGG	4
S-R14	GTCTCGTGGGCTCGGAGATGTGTATAAGAGACAGCCAACCAGAAGTGATTGTACCC	4
